# 3D Gaze Estimation Using RGB-IR Cameras

**DOI:** 10.3390/s23010381

**Published:** 2022-12-29

**Authors:** Moayad Mokatren, Tsvi Kuflik, Ilan Shimshoni

**Affiliations:** The Department of Information Systems, University of Haifa, Mount Carmel, Haifa 3498838, Israel

**Keywords:** eye tracking, 3D gaze estimation, corneal imaging

## Abstract

In this paper, we present a framework for 3D gaze estimation intended to identify the user’s focus of attention in a corneal imaging system. The framework uses a headset that consists of three cameras, a scene camera and two eye cameras: an IR camera and an RGB camera. The IR camera is used to continuously and reliably track the pupil and the RGB camera is used to acquire corneal images of the same eye. Deep learning algorithms are trained to detect the pupil in IR and RGB images and to compute a per user 3D model of the eye in real time. Once the 3D model is built, the 3D gaze direction is computed starting from the eyeball center and passing through the pupil center to the outside world. This model can also be used to transform the pupil position detected in the IR image into its corresponding position in the RGB image and to detect the gaze direction in the corneal image. This technique circumvents the problem of pupil detection in RGB images, which is especially difficult and unreliable when the scene is reflected in the corneal images. In our approach, the auto-calibration process is transparent and unobtrusive. Users do not have to be instructed to look at specific objects to calibrate the eye tracker. They need only to act and gaze normally. The framework was evaluated in a user study in realistic settings and the results are promising. It achieved a very low 3D gaze error (2.12°) and very high accuracy in acquiring corneal images (intersection over union—IoU = 0.71). The framework may be used in a variety of real-world mobile scenarios (indoors, indoors near windows and outdoors) with high accuracy.

## 1. Introduction

For most people, when we want to obtain information about an object in our surroundings, we first look at it. Eye contact is the most natural way for most people to interact with their surroundings. Hence tracking users’ eye gaze is a common way to understand where their attention is focused. Eye tracking has applications in many areas, including medicine [1], psychology [2], driver testing [3], human–computer interaction (HCI), usability research [4] and more. The advancement made in the development of mobile eye tracking technology have also enabled researchers to use eye gaze as an indicator of human visual attention in mobile scenarios. One specific example of the use of a mobile eye tracker identifying the focus of attention (e.g., using gaze as an intuitive pointer) was as part of a museum visitors guide system where the gaze served as an input modality for controlling the system [5]. In a follow-up study, the eye tracker-based system combined with hand gestures as a second input modality for controlling the system was presented in [6]. In other areas, mobile eye tracking was used in augmented reality applications [7], in research focusing on cockpits [8] where eye tracking data were collected and analyzed in the challenging environment of the commercial airline flight deck, and more.

Notwithstanding the advances in eye tracking technology, especially the improvements in video-based oculography (VOG), the most commonly used technique, many factors still affect the technology’s performance, which limits its use in daily life. One major challenge is the need for a calibration procedure before eye tracking can be initiated and the need for re-calibration whenever the device shifts even slightly. Another challenge is dealing with lighting conditions and reflections that can affect the estimation accuracy drastically [9]. The calibration must be done on a per user basis [10], because the eye tracking raw data must be transformed into gaze points in the real world and it should be done individually per user since users’ facial structure varies between users as does the placement of the head-mounted device vis-a-vis the eye. The calibration is done by looking at predefined points, usually markers on a screen, from a predefined position. There should be a sufficient number of points on different parts of the screen, and every point must be presented for enough time to allow sufficient raw data to be collected. The process is time consuming, and when it comes to mobile eye trackers, where the device is a wearable headset, any small movement of the device means that it must be re-calibrated [11].

Several studies used different techniques to try to overcome the calibration challenge. Alnajar et al. [12] suggested using gaze patterns of humans who previously observed a particular scene as an indicator where a new user will look while viewing the same scene. They achieved a gaze error of >4°. Another study [13] offered an online learning algorithm that uses mouse-clicked positions to infer where on the screen the user is looking. Each mouse click triggers a learning sample acquisition, but a cursor and its positions must be used to collect a sample of calibration points. The authors achieved an average accuracy of 2.9°. In one work [14], users’ hands and fingertips were used as calibration samples. Users only had to point with their finger to various locations in the scene. The proposed approach achieves comparable accuracy to similar marker-based calibration techniques but still requires users to look at specific points in the scene. In this study, an average accuracy of 2.68° was achieved. More recent work [15] used an RGBD front camera and saliency-based methods to auto-calibrate a head-mounted binocular eye tracking system. They achieved an accuracy of 3.7° indoors and 4.0outdoors.

A thought-provoking analysis of what information is embedded within a single image of an eye was presented in [16]. Their key observation is that the combination of the cornea of an eye and the camera capturing the appearance of the eye can be viewed as a catadioptric imaging system, a corneal imaging system. To calibrate the system, they used a geometric model of the cornea to estimate its 3D location and orientation. In another work [17], the corneal image was used to connect with the environment. They used two eye cameras to estimate the gaze vector using a simple homography that assumes that the eye is a 2D surface, whereas in reality, it is a 3D object (the eyeball).

In this work, following Nishino and Nayar [16] and Lander et al. [17], we aim at estimating the gaze vector based on the corneal imaging system and using a 3D model of the eye. Our goal was to develop a mobile, user-unaware, seamless gaze estimation system that can be used in real word scenarios. We want to be able to infer what the user is looking at, at every moment, while avoiding the need for an explicit calibration. The mobile eye tracker we use is a headset consisting of two eye cameras (IR and RGB cameras) and a front scene camera. The goal is to use the eye cameras to estimate the gaze direction and acquire corneal images, and then use the front scene camera to capture the real-world scene. Our objective is to calibrate all the cameras, more specifically to compute the mapping transformation between camera points and most importantly to do this automatically and reliably without involving the user. Achieving calibrated cameras without asking the user to look at objects, or asking them to perform special requests, will allow the process of calibration to be transparent and unobtrusive to the user. For that, sample images from IR and RGB cameras are continuously taken in the background while the user is walking around, then pupil center points are computed using deep learning algorithms and finally 3D model of the eye is computed and used for computing the 3D mapping transformation. The user can inadvertently move the glasses on their face, making the model inaccurate. Thus, from time to time the model is evaluated and if not accurate enough the process is repeated again without the user being aware of it.

We want to be able to transform, at every moment each point, especially the pupil center, from the eye camera to the scene camera. The idea is to use image matching between the corneal image and the scene image in order to compute a mapping transformation between the cameras. The challenge is that changes in lighting conditions can harshly affect the process of detecting the pupil area in the RGB camera dramatically. The use of two eye cameras enables us to continuously and reliably track the pupil using the IR camera, while employing the RGB camera to acquire corneal images for computing a mapping transformation with the scene camera. Both eye cameras build a stereo vision system that is used to compute the 3D geometric model of the eyeball. Once the 3D model is computed it is used to compute 3D transformation between the IR camera and the RGB eye camera. As detecting the pupil in the RGB eye camera is hard and challenging, we first detect the pupil in the IR camera - an easy and reliable operation; we then transform it using the computed 3D transformation for the RGB eye camera. Later, the gaze point will be transformed to the scene camera using image matching.

Traditionally, studies on eye tracking development devices evaluate their performance in lab conditions [18,19,20]. Here we exit the lab and develop a framework for 3D gaze estimation that works in the real world. It was evaluated in the real-world scenarios experiments such as when walking indoors, being indoors near windows, and outdoors.

Herein, we demonstrate and evaluate a framework that integrates the following components:

- Computing reliable stereo mapping between the IR to the RGB eye cameras using a 3D model of the eye.
- Computing the 3D gaze directions.

The framework was evaluated in a user study in a realistic scenario and generated encouraging results.

## 2. Eye Tracking Techniques

Several techniques exist for eye tracking and gaze estimation. In the past electro-oculography was used to measure eye movements by placing electrodes around the eye [21]. Presently, video-based techniques (employing cameras) are the main techniques used to detect eye movements, compute gaze directions and map these to the world in order to identify the user’s focus of attention. These techniques are standard in both stationary eye trackers and mobile (head-mounted) eye trackers. They are discussed and reviewed in [22].

**Figure 1:**
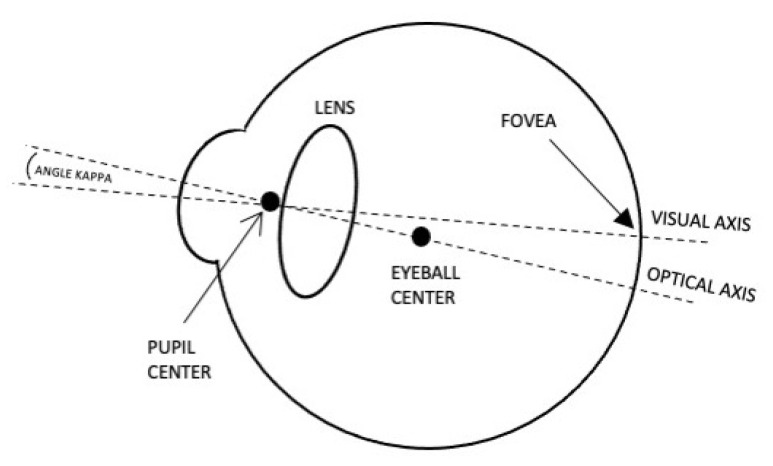
Human eye visual axes. Both visual and optical axes pass through the pupil center. Only the optical axis can be computed.

Detecting and tracking the pupil is an essential step toward developing a mobile eye tracker, as the pupil center may be used to indicate the actual gaze point in the real world. The eye has two main axes, visual and optical, as illustrated in Figure 1. The visual axis is defined as the line passing through the fovea and the pupil center to the world. It is also defined as the line of sight (LoS) and determines the real gaze direction. On the other hand, the optical axis is defined as the line passing through the eyeball center and the pupil center out to the world (3D gaze vector). The angle kappa is defined as the angle between the visual axis and the optical axis, differing from one person to another. Additional visual axes, definitions and the angles between them can be found in [23]. All eye tracking techniques use the optical axis for gaze estimation.

To develop a camera-based eye tracking system, having a way to detect the pupil reliably and continuously is crucial but has yet to be conclusively found [22,24]. Several eye detection techniques were reviewed in Hansen and Ji [24], who discussed the various challenges, such as occlusion by the eyelids, degree of openness of the eye, head pose and more, which must be overcome to achieve a definitive eye tracking method. The most frequently used eye detection methods at present are infrared (IR) cameras. Several shape-based methods for detecting the pupil in IR images exist [18,25,26,27,28]. All perform well, but when it comes to real world challenges such as light reflection and changes in illumination and occlusions, the detection rate decreases. To overcome these challenges, deep learning algorithms are now being used to robustly detect the pupil; see [29,30,31,32].

Gaze estimation techniques can be divided into two types, based on the dimensions they use either 2D or 3D. Most previous works used 2D gaze estimation (e.g., [18], where the center of the pupil in the eye image is considered the gaze point and is transformed into the world camera image). Several studies have used 3D gaze estimation in real world settings where the gaze is computed as a 3D vector and transformed to a point in the image of the world camera [33,34]. Existing gaze estimation methods are further divided into two categories, model-based methods and learning-based (appearance-based) methods. Model-based methods such as that offered in [35] exploit the geometric model of the eye to estimate the gaze. Learning-based methods learn a mapping transformation from the eye image to the gaze estimation and usually do not require individualized calibration. Several techniques for appearance-based gaze estimation were proposed and presented in [36,37,38,39], all of which require prior knowledge about the scene.

## 3. Framework for 3D Gaze Estimation System

In this section, we present our framework for 3D gaze estimation. The calibration procedure is performed automatically and individually for each user before use (and only this once). It is an unobtrusive process and the user is unaware of the calibration process when it occurs and is not instructed to look at specific objects. All that the user needs to do is to behave normally.

**Figure 2:**
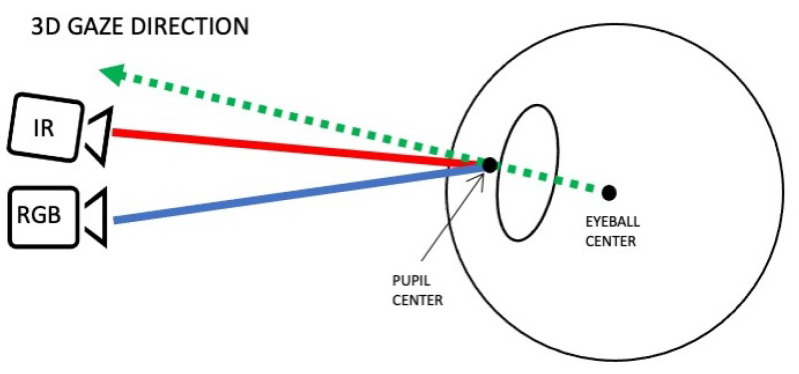
Illustration of 3D gaze direction mapping using a 3D model of the eye. The green ray is the 3D gaze direction.

In our study, we use a head-mounted device comprising of three cameras: a scene camera and two eye cameras, one infrared (IR) and one RGB. The IR camera is used to reliably track the user’s pupil. A deep learning algorithm is used to detect the pupil’s center as seen by the IR camera, whose position is then transformed into a position in the RGB image plane using stereo mapping (3D transformation). The role of the mapping is to transform the point in the IR camera image plane, into a point in the RGB camera image plane. The primary function of the RGB eye camera is to acquire corneal images around that point, which can be matched to points in the scene image and identify the user’s point of interest. A small number of RGB images in which the pupil has been detected are used for computing a transformation between the eye cameras and the front scene camera. The use of three cameras instead of two cameras (an eye camera and a scene camera, which is more typical) enables us to compute the 3D gaze direction, starting from center of the user’s eyeball and passing through it to the world (see Figure 2 for an illustration of the concept). To do this, stereo vision (two eye cameras) is used to model the eyeball. Later on, the 3D gaze direction will be transformed into a point in the image of the scene camera.

Sample points on the pupil center detected in both types of images are used to build a 3D model of the user’s eyeball. This model is then used to compute the 3D transformation between the two eye cameras and compute the 3D gaze direction. Thus, given a detected pupil in an IR image, its position in the RGB image is computed and the corneal image is extracted from around it.

### 3.1. Deep Learning for Pupil Detection

**Figure 3:**
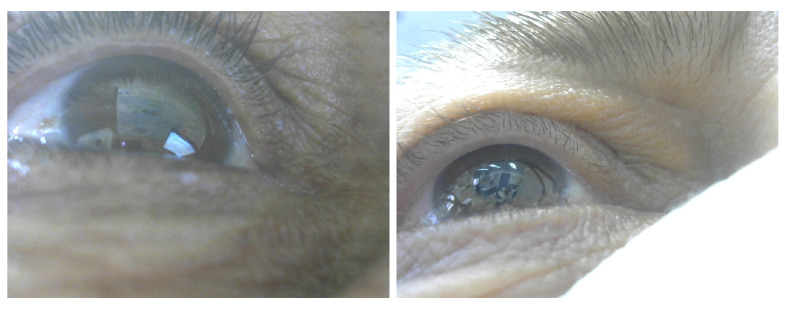
**Left**: RGB image with contact lenses, **right**: RGB image (without contact lenses) with light reflection.

Detecting and tracking the pupil continuously and reliably is crucial in building eye tracking systems. Detecting the pupil in IR images is quite easy, given that the contours of the pupil are salient. As noted earlier, previous works used model-based and learning-based approaches. Both techniques work well for detecting the pupil in IR images. When it comes to RGB images, challenges arise. The lighting conditions and reflections can drastically affect the process of detecting the pupil, and in some cases when there is low or strong lighting, the pupil region is not salient (see Figure 3, right, for an example). Hence detecting the pupil in an RGB image using model-based technique fails [40]. Training a deep learning algorithm for pupil detection in RGB images can overcome the challenges [29]. Even with deep learning methods, however, detecting the pupil in RGB images is challenging. It becomes even more so when the iris color is dark, since the pupil contours are not salient (see Figure 3, right, for an example).

For this work, we trained an existing faster region based convolutional neural network (RCNN) for object detection [41] for the purpose of detecting pupils in both IR and RGB camera images. The CNN is based on a region proposal network (RPN), which takes an image (of any size) as input and outputs a set of rectangular object proposals, each with an objectness score. We trained the RCNN twice for each image type (IR and RGB), each with 960 training images taken from 18 participants (total of 1920 images). The pupil regions in the training images were manually labeled. Given an image of the eye in a test procedure, we consider the highest scoring bounding rectangle (if such exists) with an intersection over union (IoU) score of at least 0.9 (if such exists) as the pupil boundary. Our detection rate on IR images is 78% indoors, 42% near windows and 46% outdoors. While on RGB images, 48% indoors, 12% near windows, and 2% outdoors.

### 3.2. Stereo Mapping

Since detecting the pupil is easy in IR cameras and hard and challenging in RGB cameras, in our approach we use the IR camera to reliably and continuously track the pupil, and the RGB camera to extract corneal images, which are also used for matching points on the images taken by the front camera in order to identify the user’s focus of attention.

The IR and RGB cameras comprise our stereo system. Given a point in the IR camera image plane, we should be able to transform it into a point on the RGB camera image plane. Normally, using 2D transformations (such as the fundamental matrix), a point is transformed into a line. In our case, we need a model in order to be able to transform a point into a point; a 3D transformation meets our needs. For computing such a transformation, the cameras’ internal parameters, as well as information about the object on which the cameras are focused (the eyeball) should be known. The average human eyeball radius, as known from the literature, is approximately 12 mm [42].

To compute a 3D transformation between the cameras in a stereo system, the relative pose between the cameras as well as the relative pose between the cameras and the human eye should be known. The relative pose between the cameras is computed using stereo camera calibration, which is an offline process that is done once and is independent of users’ features. In our system, the calibration procedure was performed using a chessboard pattern with a cell size of 2mm according to the technique proposed in [43]

The device we use here has an eye camera mount that can be adjusted to align with the shape of the user’s face. Obviously, the distance and the angle from the eyeball to the camera differs from person to person. This means that the relative pose between the cameras and the eye must be computed individually per user. Previous work [44] compared several 2D and 3D models used for mapping pupil centers. Even though all of the models are good, but the 2D models are not applicable for our work in this framework. We need the 3D direction of the gaze, hence a 3D transformation. For this to be done, the eyeball model is built individually for each user and used when computing a 3D-based mapping between the cameras.

**Figure 4:**
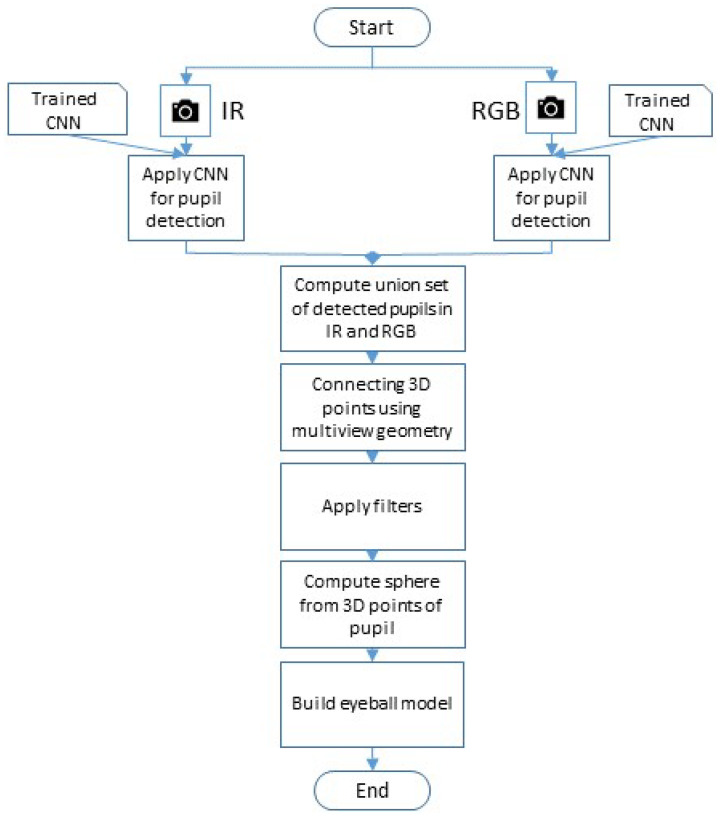
The flowchart of 3D geometric model building using two eye cameras.

The flowchart presented in Figure 4 lays out the process of building the 3D model used for computing the 3D transformation:

1. First, two sets of images from the IR and RGB cameras are captured simultaneously.
2. Thereafter, deep learning algorithms are applied on each set, accordingly, to extract the center of the user’s pupil.
3. A set of pairs of images of the detected pupils, from both cameras, are computed.
4. 3D points corresponding to the pupils’ centers are computed and connected using the cameras’ parameters and multi-view geometry.
5. Outliers are filtered out.
6. Finally, a 3D model is built by computing a sphere from the 3D points.

### 3.3. Eyeball Modeling

To build a 3D model of the eyeball for a specific user, sample points from both cameras are needed. To this end, images of the eye are taken simultaneously by both cameras. We collect points from pupil boundaries detected by the deep learning algorithm. More specifically, considering the pupil’s bounding rectangle, we use the top left, center, and bottom right points.

Given a stereo camera system and a 3D point in the space, the projection of that point to the image plane of each camera creates a ray from the 3D point to the image plane. From the cameras’ point of view, the intersection point of these two rays is the 3D point that lies on the eyeball. Hence, given a set of corresponding points in the two image planes, looking at the intersections of the projected rays of the corresponding pair, we obtain a set of 3D points. We know that these points lie on the eyeball surface, so if we have enough 3D points, the center of the eyeball can be found since the radius is known (12 mm).

In the following sections we denote the IR camera as $C_{ir}$ and to the RGB camera as $C_{rgb}$. Both cameras and the eyeball (especially the eyeball) lie in a 3D space. We set $C_{ir}$ to be at the origin of the coordinate axes ($C_{ir}=\left[0,0,0\right]$) and its rotation matrix as *I*. The relative orientation of $C_{rgb}$ is *R* and its translation vector is *T*. Hence the position of $C_{rgb}$ relative to $C_{ir}$ is l=−RT. Moreover, $K_{ir}$ is the intrinsic matrix of $C_{ir}$ and $K_{rgb}$ of $C_{rgb}$. Therefore, all the coordinates in the space are relative to $C_{ir}$.

#### 3.3.1. 3D Points in Space

Let $initSet_{ir}$ and $initSet_{rgb}$ to be the extracted sample points from both cameras, respectively. Let,

$$p_{i}\in initSet_{ir},$$

$$p_{i}^{\prime }\in initSet_{rgb}$$

be sample points on the cameras’ image planes. The direction vector of the projection $p_{i}$ in the real world space is

$$N_{i}=K_{ir}^{-1}p_{i}$$

and the direction vector of the projection $p_{i}^{\prime }$ in the real world is

$$N_{i}^{\prime }=R\left(K_{rgb}^{-1}p_{i}^{\prime }\right).$$

We first normalize $N_{i}$ and $N_{i}^{\prime }$. Then we compute the 3D point in space that is the intersection point between the two rays

$$P_{ir}\left(\alpha_{ir}\right)=C_{ir}+\alpha_{ir}N_{i},$$

$$P_{rgb}\left(\alpha_{rgb}\right)=C_{rgb}+\alpha_{rgb}N_{i}^{\prime },$$

where $\alpha_{ir}$ and $\alpha_{rgb}$ are the respective distances between the camera centers to the intersection point.

We solve

$$C_{ir}+\alpha_{ir}N_{i}\approx C_{rgb}+\alpha_{rgb}N_{i}^{\prime }$$

as follows:

$${\left[N_{i},N_{i}^{\prime }\right]}^{T}\left[\alpha_{ir},-\alpha_{rgb}\right]\approx \left[-C_{ir},C_{rgb}\right].$$

$\left[\alpha_{ir},-\alpha_{rgb}\right]$ are found using the linear least squares method. Thus,

$$S_{ii^{\prime }}=\frac{P_{ir}\left(\alpha_{ir}\right)+P_{rgb}\left(\alpha_{rgb}\right)}{2}$$

is the middle point between the two closest points, which is the estimated 3D point of the pupil center in space and

$$d_{ii^{\prime }}=\|P_{ir}\left(\alpha_{ir}\right)-P_{rgb}\left(\alpha_{rgb}\right)\|$$

is the distance between the two estimates.

Under realistic conditions in a noisy environment, these vectors will not intersect, but will be close enough to each other. We set the maximum distance $d_{ii^{\prime }}$ between the vectors to be 2mm in order to be considered to be intersecting as defined in [44]. Moreover, we compute the median of all the ray’s norms $\left\|\alpha_{ir}N_{i}\right\|$ and $\left\|\alpha_{rgb}N_{i}^{\prime }\right\|$, and accordingly we filter out the corresponding 3D points of the sample points $p_{i}$ and $p_{i}^{\prime }$ that are out of range ±2 mm from the computed median. Filtering out point pairs that do not satisfy these conditions is done to detect pairs where an error in the detection of the pupil by either cameras occurred, or when the concurrent capture process in the two cameras fails. The 3D model is built using the filtered points only.

#### 3.3.2. 3D Model Computation

The stereo pair of cameras (IR and RGB) faces the eye. The eyeball equation, under the assumption that the eyeball is spherical, is:

$$\left\|P-C\right\|=r^{2},$$

where *P* is a 3D point, *C* is the center of the sphere and the known radius is *r*. The problem is to find the center of the sphere.

Let *S* denote the set of the pupils’ 3D points. The next step is to compute the equation of the sphere. We should note that *S* may contain false positive points that do not lie close to the sphere, since the pupil detection algorithm may have generated errors, and as a consequence, the eyeball estimation may be affected. Some of the false-positives are removed using the 3D point filtering methods described earlier, but not all of them. To remove any remaining false-positives entirely, the eyeball reconstruction process is divided into two phases, approximation and optimization. We first approximate the center of the eyeball as follows. We compute the plane on which the points in the set *S* lie. The plane is tangent to the sphere with radius *r*, so we look at the normal of the plane and add *r* to it. The result is an approximation of the center of the sphere. We note that this approximation is used only as a starting point in the second stage, the optimization phase.

Formally, let $\bar{P}$ be the average point in *S*, the center of sphere (eyeball) *C* be:

$$C=\bar{P}+Nr,$$

where *r* is the eyeball radius, and *N* be the normal to the plane of the sphere, which is computed using $SVD(S-\bar{P}).$

After approximating the center of the sphere, we run an optimization procedure using an M-estimator [45], a robust estimation technique that is widely used [46]. This is done using the following robust distance function.

$$dist\left(P,C,\delta \right)=\left\{\begin{array}{cc} |\left\|P-C\right\|-r|, & \mathrm{if}\;|\left\|P-C\right\|-r|\leq \delta \\ \delta , & \mathrm{otherwise} \end{array}\right.$$

The goal here is to minimize the error caused by the approximation in the first phase. To do this, we want to find the center of a sphere that minimizes the sum of distances between all the 3D points (that may contain outliers) and the surface of the sphere. We run two M-estimators, first with δ=1 to obtain a better approximation of the center and then with δ=0.5.

Finally by computing the center of the sphere and knowing the eyeball radius, the eyeball is modeled.

### 3.4. 3D Mapping Transformation

In our framework, after building the 3D model, we only need to track the pupil with one camera (the IR camera). This is done continuously and reliably using a deep learning algorithm. Then point-to-point transformation from the IR image plane to the RGB image plane is computed. In addition, the 3D gaze ray is computed by defining a vector starting from the eyeball center, passing through the computed pupil center to the outside.

Every so often, we can use the data from two cameras to validate the 3D model, and if there is a problem, we can rebuild and update the model. To do this, occasionally we try to detect the pupil using the RGB camera, even though it is not needed for the transformation computation. We compare the pupil center in the RGB camera transformed from the IR camera with the pupil center detected by a deep learning algorithm. If there are large distances for a certain period of time, we can infer that the 3D model is not valid anymore (for instance, due to movement of the headset), and a new model should be built.

Given a point (pupil center) in the $C_{ir}$ image plane, we want to find which point in the $C_{rgb}$ image plane corresponds to it. Let,

$$p_{ir}\in C_{ir}$$

be a point in the $C_{ir}$ image plane and let *C* be the center of the eyeball. The projection of the point in the space is a ray with vector direction,

$$v=K_{ir}^{-1}p_{ir}$$

which has been normalized. We want to know which point in this direction (ray) lies on the sphere. Formally, we are searching for α that satisfies:

$${\left(\alpha v-C\right)}^{2}-r^{2}=0$$

where *C* is the eyeball center and *r* is its radius. There exist at most two roots that satisfy the equation. We will choose the smaller value if two roots do exist, as this represents the nearest (first) intersection point with the eyeball surface. If the equation has no solution, we approximate it by taking the real part of the roots (a complex number). Due to noisy points and imperfection(s) of the eyeball model, we argue that one root will never exist as this would mean that the ray is tangent to the eyeball.

Let, $P_{w}=\alpha v$ be the recovered 3D point in space that lies on the sphere. What remains to do is to transform $P_{w}$ into a point on the $C_{rgb}$ image plane. This is done using the perspective transformation of $C_{rgb}$. Formally, we define *M* to be the perspective transformation,

$$M_{rgb}=K_{rgb}\left[R\;|\;T\right].$$

Accordingly, the corresponding point $p_{rgb}$ of $p_{ir}$ in the $C_{rgb}$ image plane is:

$$p_{rgb}=M_{rgb}\left[P_{w}\;1\right],$$

where $p_{rgb}$ lies on $C_{rgb}$.

### 3.5. Model Transformation Process

**Figure 5:**
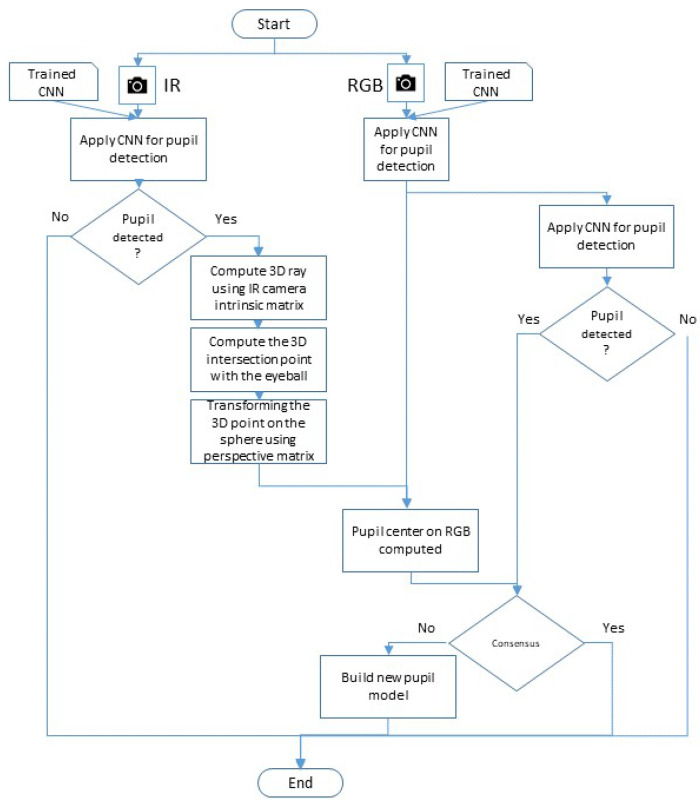
The flowchart for the point-to-point model transformation using the IR and RGB eye cameras.

To clarify and simplify the description of the 3D mapping transformation, we summarize the process in the flowchart in Figure 5. The process is divided into two real-time sub-processes that run simultaneously: (1) transforming the pupil center from the IR image plane to the RGB image plane (left side); and (2) model validation (right side).

1. Given an image from the IR camera, a deep learning algorithm is applied to detect the pupil center. If the pupil is detected, the 3D ray to the real world is computed. The ray is used to compute the intersection point with the eyeball. Finally, the intersection point is transformed from the IR image plane to the RGB image plane using the perspective projection matrix $M_{rgb}$.
2. To validate the model, we occasionally verify the data. More specifically, we verify the transformed points from the IR camera. To do this, once in a while we try to detect the pupil center in the RGB image using a deep learning detection algorithm and check for agreement with the transformed pupil center point from the IR camera. We expect such agreement. If, however, there is no consensus after several continuous images, we can infer that the model is not valid anymore and a new eyeball model is needed.

### 3.6. Self-Supervision for Future Deep Learning Algorithms

We now discuss how we can automatically generate labeled datasets to be used for training future deep learning pupil detection models. Previously, we described how we detect pupils by training a network using hand labeled datasets of pupils. Obviously, manually labeling images is exhausting and time consuming. Having an automatic tool for generating automatic and reliable labeled pupils in eye images used for self-supervision of deep learning algorithms is a big step toward developing future eye tracking system in general, and training reliable deep learning algorithms for detecting pupils in particular. Foremost, self-supervision will improve future deep learning algorithms’ detection of pupils in RGB images.

The idea is that, while the users use our eye tracking system, we store the RGB images together with the computed pupil boundaries. More specifically, given an IR image, we apply the pupil detection algorithm. Then, considering the bounding rectangle, we transform the top left and bottom right points into the RGB camera space, which define the pupil’s region in the RGB camera. To ensure that we build a reliable and high-quality automatic labeling procedure, we compare each computed pupil center in the RGB image with the detected center using the object detection algorithm. We define the agreement between the pupil regions (the one computed using 3D, and the one computed using deep learning for RGB) to be an IoU score above 0.5. If the pupil cannot be detected in the RGB image, or if the agreement score is low, we do not store the image with the label. As a result of this process, a large number of new training data examples are obtained.

## 4. Experiments

**Figure 6:**
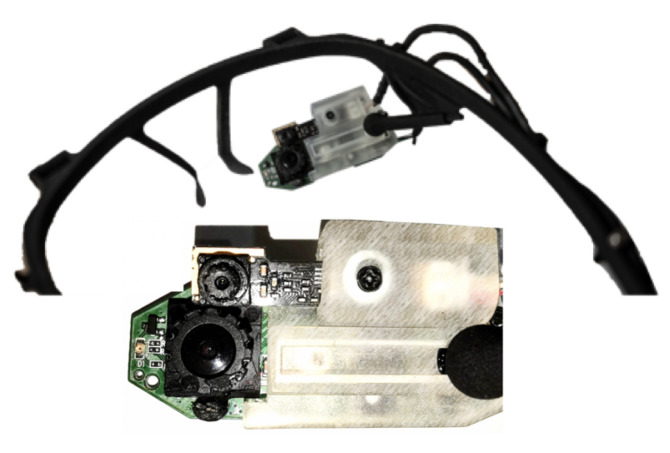
Head-worn corneal imaging glasses and zoomed in view. The upper (small) camera is the IR camera, and the lower is the RGB camera.

To evaluate our framework for the 3D gaze estimation, we developed a prototype that automatically calibrates the system without requiring any help from the users. Users wear the head-mounted device presented in [17] (see Figure 6), consisting of two eye cameras, an IR camera and an RGB camera. The IR camera continuously and reliably tracks the user’s pupil, while the RGB camera is used mainly for acquiring corneal images. The framework builds a 3D model of each user’s eye, computes robust and reliable 3D transformations between the eye cameras, and computes the user’s 3D gaze direction in the real world. The process of building the 3D eye model is done automatically, without the user having to do anything but wear the device. The process uses deep learning techniques to detect the pupil in the IR and RGB images, and using the known parameters of the eyeball.

### 4.1. Tools and Methods

The prototype was developed using MATLAB and Python. The training procedure for the pupil detection deep learning was performed using Python and the TensorFlow API for object detection [47]. The cameras’ capture process and the framework implementation were implemented using MATLAB while integrating the trained pupil detection deep learning model. The cameras’ capture tool runs two parallel processes for capturing images from both cameras simultaneously at a frame rate 20 fps. To avoid so many duplicates in the evaluation process, we need only 1 fps, and most importantly a synchronized couple of images (from IR and RGB). For that, at every second, we choose the couple with the closest timestamps. The framework implementation includes building a 3D eye model, computing the 3D mapping transformation, extracting corneal images and computing 3D gaze directions. The 3D gaze direction was computed and defined by a 3D vector starting at the center of the eyeball and passing through the pupil center to the real world. This definition is an approximation of the real human visual system, where the gaze vector starts from the fovea and passes through the cornea center out to the real world. As the kappa angle, which plays a key role in defining the visual axis differs between people and cannot be measured in standard ways, our approach is a good approximation.

### 4.2. Experimental Procedure

We conducted an experiment in a realistic setting at the University of Haifa campus. Twelve participants (6 males, 6 females) took part in the experiment. One participant wore contact lenses (see Figure 3 left). The experiment was intended to evaluate the feasibility and accuracy of our system under different conditions. Participants, monitored by one of the researchers, wore the mobile headset that was connected to a laptop, and used the system. The experiment was conducted in three sessions, indoors, indoors near windows and outdoors. Participants were instructed to behave normally, gaze naturally and walk freely during each session. The only request was not to move the headset, which meant that participants tried not shift the headset during the three sessions. The free movement lets us focus on evaluating the accuracy of the system in all situations and under all conditions.

The experiment took about 30 min. This period included the participates receiving an introduction and a description of the research and its goals, allowing about 7 min for each session, and a couple of minutes for moving between different sessions (such as going from inside the building outside). During each session, 150 images from both cameras were taken simultaneously using the cameras’ capture tool, one image per second, saved to the laptop’s RAM and transferred to its disk at the end of the session for synchronization purposes. Saving 150 images (1280 × 960) on the laptop’s hard disk using MATLAB took about 4 min (MacBook Pro 2017).

**Figure 7:**
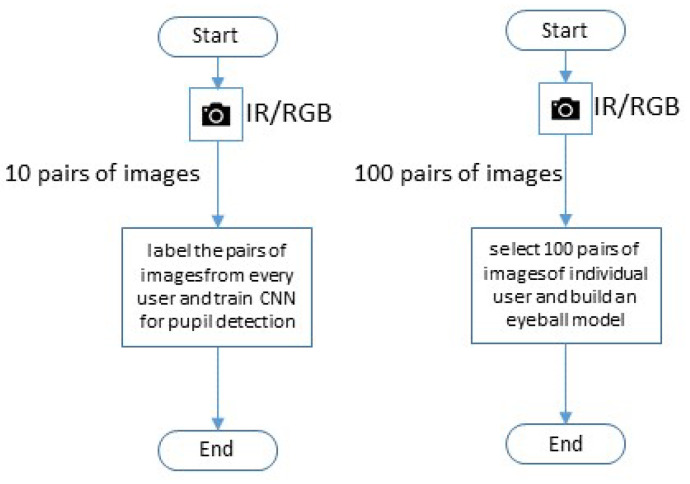
The flowchart for the model describing the procedure of choosing test images.

From each session, we used 10 pairs of images for training the deep learning model. In addition, from the indoor sessions only, we used 100 pairs of images to compute the 3D eyeball model; the remainder were used as test pairs of images for evaluation. Figure 7 illustrates the process of image capture.

### 4.3. Evaluation

The main parts of the evaluation assess the accuracy of the computed mapping transformation and the corneal image extraction, and to rate the 3D gaze error. For this, we used the test pairs of images captured during the experiment, each pair consisting of IR and RGB images. For each participant, 100 pairs of images were used for modeling the eyeball as a first step, and the 3D mapping transformation was computed accordingly. From each IR image in every test pair, we tried to detect the pupil. If the pupil was detected in the IR image, it was transformed automatically into an RGB image. For the purpose of comparison, we labeled the pupil in each RGB image in every test pair manually, which is the closest way to reach the ground truth. We used the Euclidean distance between the automatically computed pupil center in the RGB image and the ground-truth (manually labeled) point as a metric for comparison.

**Figure 8:**
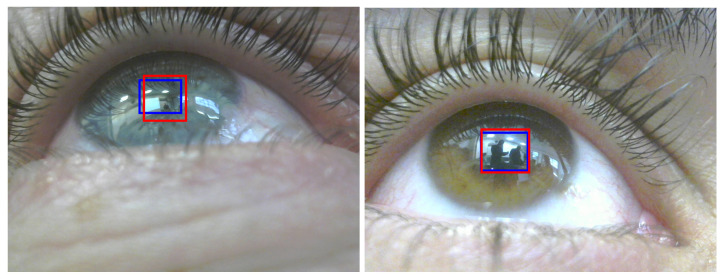
Automatic detection (red) vs. manually labeled (blue) of pupils. **Left**: *IoU* = 0.6, **right**: *IoU* = 0.84.

In addition, from every IR image in each test pair, the pupil’s bounding rectangle was computed using two points (top left and bottom right). Each bounding rectangle in the IR image was transformed into a bounding rectangle on its RGB image pair using a two-point 3D transformation. The transformed bounding rectangle in the RGB image is the corneal image. We compared the extracted corneal image with the ground truth and used the IoU (Jaccard index) defined by the area of overlap/area of union as a metric for comparison. Normally IoU>0.5 is considered to be a good prediction, Figure 8 demonstrates this with a good IoU (0.6, left image) and a very good IoU (0.84, right image).

**Figure 9:**
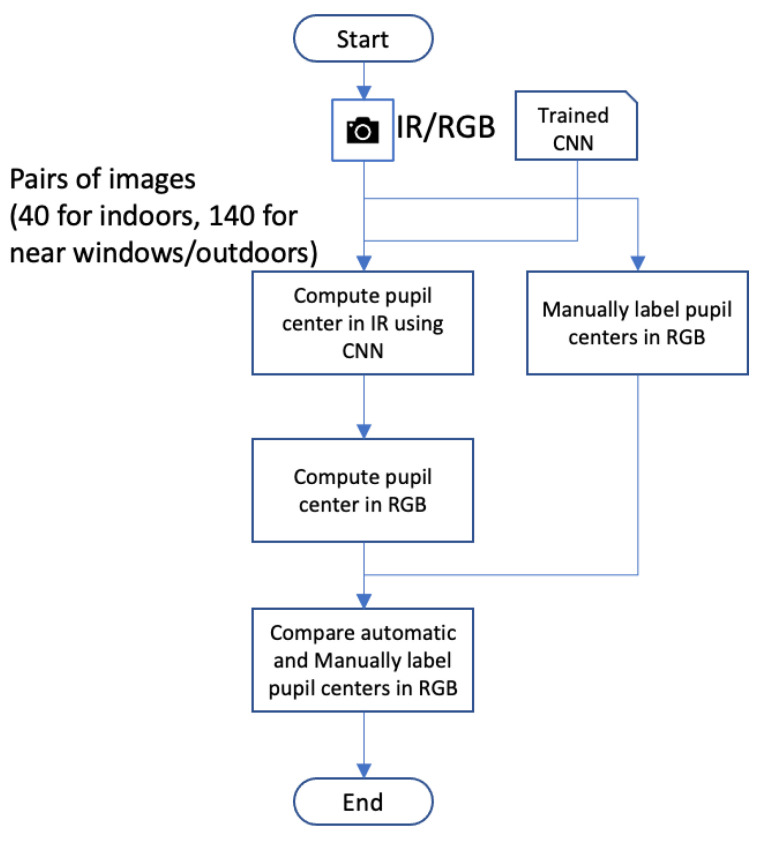
The flowchart describing the procedure of the framework’s evaluation.

For each pupil center pair in the RGB image (computed and manually labeled), we also compared the automatically computed 3D gaze using the manually labeled center and used the angle between vectors as well as the Euclidean distance (in pixels) between pupil centers as a metric for comparison. Figure 9 illustrates the comparison process.

### 4.4. Results

**Figure 10:**
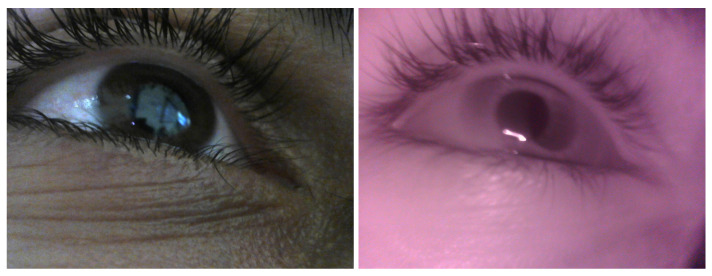
Unsynchronized capture. Left: RGB, right: IR image where the pupil is distorted during a saccade.

Twelve participants including students and staff from the University of Haifa took part in the study. Each participated in three sessions, for a total of 36 sessions. The data collected in seven sessions were invalid: three sessions for one participant, and one session (indoors) for another participant were unsynchronized. Specifically, the synchronization algorithm between the IR and RGB cameras did not perform correctly. As a result, each pair of images was not taken at the same timestamp (see Figure 10 for an example). Note that for the latter participant, the 3D model was built using the indoor session, even though it was invalid, as the data were filtered when building the 3D model. Moreover, in three sessions for another participant, the deep learning algorithm for RGB pupil detection performed poorly, which directly affected the model building process.

For each pair of test images (IR and RGB), we define each pair as a valid test pair if the pupil in the IR image was detected, and if the pupil in the RGB image had been manually labeled. The resulting metrics are: (1) gaze error in degrees when considering the 3D gaze direction; (2) gaze error in pixels; (3) percent of consensus between pupils’ bounding rectangle on the RGB image between the automatic method and the deep learning algorithm; (4) IoU of pupils’ bounding rectangles between automatic method and the ground truth; and (5) IoU of pupils’ bounding rectangle between the deep learning algorithm and the ground truth.

For each metric and for each participant, we calculated the median to filter out outliers measurements. Finally, we calculated the median of all participants’ medians (median of medians). Table 1 summarizes the results.

We also applied each metric to each session type. In addition to the previously defined metrics, we also calculated, for each session type, the percentage of pupils successfully detected by the IR camera, and the percentage of valid test pairs. Table 2 summarizes the results.

During the study, we encountered special cases (valid and invalid). Valid sessions include a participant whose iris was dark, i.e., where the pupil region is unsalient in RGB images (see Figure 3, right, for an example), and sessions captures for one participant who wears contact lenses. Invalid sessions include unsynchronized capture and light reflection on the eye. Table 3 summarizes the results for the special cases.

Looking at Table 1, there were 29 valid sessions in which: (1) the median 3D gaze error was 2.12 degrees; (2) the median gaze error was 10.04 pixels; (3) 30% of the deep learning algorithm pupil detections in the RGB images were confirmed by the transformed bounding rectangle from the IR images; (4) the median IoU between the computed bounding rectangle in the RGB and the ground truth was 0.71; and (5) the median IoU between the computed bounding rectangle in the RGB using the deep learning algorithm and the ground truth is 0.71.

Looking at Table 2, for example, in indoors sessions the pupil was detected in 78% of the cases and 70% of the test pairs were valid. Moreover, 62% of the deep learning algorithm pupil detections in the RGB images were confirmed by the transformed bounding rectangle from the IR images.

## 5. Discussion

The framework for 3D gaze estimation system was evaluated in a user study where the participants were asked to walk and look freely at objects in a mobile scenario. The study included three different sessions: indoors, indoors near windows and outdoors, which are typical mobile scenarios. The indoors scenario is a standard one where the lighting is usually fixed; indoors near windows is a special case where the reflections coming from the windows are reflected directly onto the eyes. For every user, the pupils in the datasets of eye images were manually labeled and used as a ground truth for the evaluation. We considered gaze errors and the IoU of pupils’ bounding rectangles between the automatic computation and the ground truth as metrics for comparisons.

The framework achieved a median 3D gaze error of 2.12° and 10.04 pixels, with an image size of 800 × 600. Accordingly, the error in pixels was 1% relative to the diagonal length. Moreover, the framework achieved a median IoU of 71% between the computed pupil boundary (corneal image) and the ground truth, which is considered very good. Additionally, the deep learning algorithm for detecting the pupil in RGB images achieved an accuracy of 69% in general compared to the ground truth.

Our proposed framework achieved very good accuracy compared to state-of-the-art methods that try to compute gaze without a need for calibration. A comparison with state-of-the-art methods can be found in Table 4. Nevertheless, our auto and non-obtrusive calibration technique does not require the user to perform special requests, compared to current methods where the user is still involved in the process, such as inferring mouse clicks on a screen, matching the user’s scene with previously viewed scene, asking the user to look at various locations in the scene, or to use special equipment for scanning the environment such as RGB-D cameras. Moreover, what makes our proposed framework extraordinary, unlike previous works, is the ability to work in different real scenarios: indoors, indoors near windows and outdoors.

Considering the three different session types, in the indoor sessions, the framework detected the pupil in the IR images in 78% of cases and the 3D gaze error was 2.12°. The deep learning algorithm detected the pupil in the RGB images in 73% of the cases with an IoU accuracy of 48%. In the indoors near windows sessions, the framework detected the pupil in IR images in 42% of the cases and the 3D gaze error was 2.03°. The deep learning algorithm detected the pupil in the RGB images in 12% of the cases with an IoU accuracy of 69%, a significant decrease in the number of detected pupils in IR images relative to the indoor (not near windows) sessions. In the outdoor session, the framework detected the pupil in IR images in 46% of the cases and the 3D gaze error was 2.32°. The deep learning algorithm detected the pupil in the RGB images in just 2% of the cases, with an IoU accuracy of 73%—a very low percentage of detected pupils in IR images relative to the indoor (not near windows) condition.

**Figure 11:**
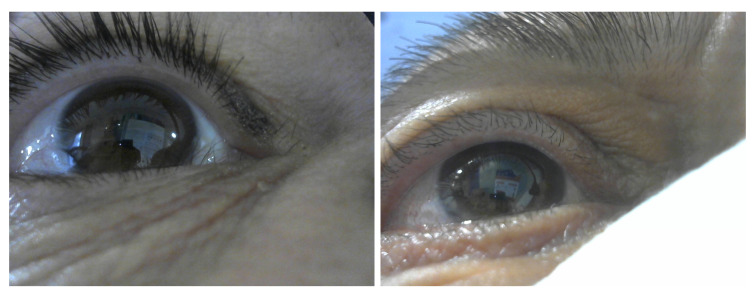
Sample images with dark iris color.

The decrease in percentage of detected pupils in RGB images (the accuracy is stable), both automatically using the deep learning algorithm and when labeled manually, is primarily caused by the limitation imposed by the reflection of the light on the eye. This limitation was noticeable during the process of manually labeling the pupils in the RGB images, where the person who tagged the test images could not see the pupil. Looking at the experiment’s special cases, the deep learning algorithm for detecting the pupil in RGB images had difficulty in dealing with dark iris color (Figure 11 for example), contact lenses and harsh light reflection. Nevertheless, in the valid cases, the framework had a low gaze error.

Despite all the technical limitations, the framework produces automatically labeled data of pupils in 30% of cases with high accuracy (IoU=0.71). The automatically generated datasets of pupils can be used to improve existing and future deep learning algorithms for detecting pupils in both IR and RGB images. In the indoor scenario, the framework produces automatically labeled images 48% of the time—an impressive achievement. In the other cases, the framework generated smaller datasets, 12% in the near windows scenario, and in the outdoor sessions, for only 2% of the cases.

After building the personal 3D model for each user, the framework uses only the IR image to estimate the gaze direction and to extract the corneal images. The framework can be used in a variety of real-world scenarios, by any user without needing a technological background, and without any special instruction and requests to calibrate the system. The framework works very well in an indoor scenario, where in 78% of the cases the pupil was detected in IR images. In the other scenarios (near windows and outdoors), the framework was less successful and this is due to the challenges posed by the lighting conditions. As shown in Table 2, there was a decrease of 14% in the number of detected pupils (from 42% to 28%) when the manually labeled pupils in the RGB images were considered. The decrease was even worse (27%) in the outdoor scenario (from 46% to 19%).

Considering the above-mentioned limitations, the main reason was the lighting reflections and the challenge of detecting the pupil in the RGB plane in these cases. It was noticeable during the manual labeling process that the pupil region is not salient (due to lack of contrast) and as a consequence some of the images in all the datasets were not labeled. One solution to the problem of unsalient pupils could be that in the future the iris region should be labeled instead of the pupil, as the contrast between the iris and the white region around is always salient. In this case, we would consider the iris center as an approximation of the pupil center. It is thus possible to use the iris center to overcome the challenges of lighting reflection and in building an accurate and robust gaze tracker [48]. Furthermore, the experiment procedure was very complex and required considerable time and effort, hence so far only 12 participants performed the study. The small number of participants eis considered to be a limitation of the study and noted in the discussion. We plan to extend the study in a future work and noted that in the future work section.

The advancement made to the development of eye tracking technology may help many studies that use the gaze as an indicator of human visual attention. Providing a mobile device that detects the human gaze can be used in different disciplines: interaction with real or virtual environments, metaverse, cockpit control and more.

## 6. Conclusions and Future Work

In this work, we presented and evaluated a framework for 3D gaze estimation using a prototype system that implemented it. The system included a mobile headset consisting of two eye cameras (IR and RGB) and a front scene camera. The IR camera is used to continuously track the user’s pupil and the RGB camera to extract corneal images. Deep learning algorithms were trained and applied for detecting the pupil in IR and RGB images. For every user, an automatic 3D model of the eye was built and used for computing a 3D mapping transformation between the eye cameras. Using the 3D model of the eye and the detected pupil center, the 3D gaze direction was estimated by computing the vector direction starting at the eyeball center and passing through the pupil center out to the real world.

The suggested framework does not require any initial calibration procedure. The user can simply put on the eye tracker on and start moving around and using it, as the calibration procedure is automatic and, as far as concerns the user, transparent. The framework was evaluated in a user study and the results are promising. We achieved a very low 3D gaze error and very high accuracy in acquiring corneal images. The framework may be used in a variety of mobile scenarios (indoors, indoors near windows, and outdoors) with high accuracy. This is a breakthrough, especially when it comes to calibration-free mobile headsets. Moreover, there is no special requirements for which cameras can be used, especially which IR sensor. In our proposed solution only adaption of the deep learning algorithms to the new sensor images is needed.

Future work will focus on improving the deep learning algorithm for pupil detection using automatically labeled pupils— a technique we presented in this paper. Moreover, we will evaluate the transformation of the 3D gaze direction to the front scene camera, and on building a fully calibration free mobile eye tracker that works in any mobile scenario.

## Figures and Tables

**Table 1:** Summary of results for valid and invalid sessions. In addition to gaze error metrics, we calculate: (1) percentage of consensus (IoU>0.5) between computed pupils’ bounding rectangle by automatic transformation from IR, and the ground truth (manually labeled) in RGB; (2) IoU between computed pupils’ bounding rectangle by automatic transformation from IR, and the ground truth (manually labeled) in RGB; (3) IoU between bounding rectangle computed by deep learning algorithm in RGB, and the ground truth (manually labeled) in RGB.

	Total No. of Sessions	Gaze Error (deg)	Gaze Error (px)	% Consensus AUTO & DL	IoU AUTO & GT	IoU DL & GT
Valid session	29	2.12	10.04	30%	0.71	0.69
Invalid session	7	4.28	15.08	28%	0.62	0.62

**Table 2:** Summary of results for valid sessions. In addition to gaze error metrics, we calculated: (1) percentage of consensus (IoU>0.5) between computed pupils’ bounding rectangle by automatic transformation from IR, and the ground truth (manually labeled) in the RGB images; (2) the IoU between computed pupils’ bounding rectangle by automatic transformation from the IR images, and the ground truth in the RGB images; (3) the IoU between bounding rectangle computed by the deep learning algorithm in the RGB images, and the ground truth in the RGB images.

Session	% Detected IR	% IR & GT RGB	Gaze Error (deg)	Gaze Error (px)	% Consensus AUTO & DL	IoU AUTO & GT	IoU DL & GT
Indoors	78%	70%	2.12	10.53	48%	0.73	0.7
Windows	42%	28%	2.03	10.02	12%	0.69	0.6
Outdoors	46%	19%	2.32	10.22	2%	0.73	0.74

**Table 3:** Summary of special cases results (valid and invalid).

Status	Case	Gaze Error (deg)	Gaze Error (px)
Invalid	Unsynchronized capture	3.71	21.45
Invalid	Light reflection	5.64	16.25
Valid	Dark iris color	3.26	16.23
Valid	Contact lenses	3.6	16.24

**Table 4:** Summary of comparison with state-of-the-art methods.

Work	Our work	[14]	[13]	[15]	[12]
Gaze error (deg)	2.03–2.32	2.68	2.9	3.7–4.0	>4

## Data Availability

Not applicable.
